# The nicotine-degrading enzyme NicA2 reduces nicotine levels in blood, nicotine distribution to brain, and nicotine discrimination and reinforcement in rats

**DOI:** 10.1186/s12896-018-0457-7

**Published:** 2018-07-24

**Authors:** Paul R. Pentel, Michael D. Raleigh, Mark G. LeSage, Thomas Thisted, Stephen Horrigan, Zuzana Biesova, Matthew W. Kalnik

**Affiliations:** 1 University of Minnesota, 100 Church St. S.E, Minneapolis, MN 55455 USA; 2 Minneapolis Medical Research Foundation, 701 Park Ave, Minneapolis, MN 55415 USA; 3 Antidote Therapeutics Inc, 708 Quince Orchard Road, Suite 250-C, Gaithersburg, MD 20878 USA; 4 Noble Life Sciences, PO Box 242, Woodbine, MD 21797 USA

**Keywords:** Nicotine, Enzyme, Metabolism, Degradation, Addiction

## Abstract

**Background:**

The bacterial nicotine-degrading enzyme NicA2 isolated from *P. putida* was studied to assess its potential use in the treatment of tobacco dependence.

**Results:**

Rats were pretreated with varying i.v. doses of NicA2, followed by i.v. administration of nicotine at 0.03 mg/kg. NicA2 had a rapid onset of action reducing blood and brain nicotine concentrations in a dose-related manner, with a rapid onset of action. A 5 mg/kg NicA2 dose reduced the nicotine concentration in blood by > 90% at 1 min after the nicotine dose, compared to controls. Brain nicotine concentrations were reduced by 55% at 1 min and 92% at 5 min post nicotine dose. To evaluate enzyme effects at a nicotine dosing rate equivalent to heavy smoking, rats pretreated with NicA2 at 10 mg/kg were administered 5 doses of nicotine 0.03 mg/kg i.v. over 40 min. Nicotine levels in blood were below the assay detection limit 3 min after either the first or fifth nicotine dose, and nicotine levels in brain were reduced by 82 and 84%, respectively, compared to controls. A 20 mg/kg NicA2 dose attenuated nicotine discrimination and produced extinction of nicotine self-administration (NSA) in most rats, or a compensatory increase in other rats, when administered prior to each daily NSA session. In rats showing compensation, increasing the NicA2 dose to 70 mg/kg resulted in extinction of NSA. An enzyme construct with a longer duration of action, via fusion with an albumin-binding domain, similarly reduced NSA in a 23 h nicotine access model at a dose of 70 mg/kg.

**Conclusions:**

These data extend knowledge of NicA2’s effects on nicotine distribution to brain and its ability to attenuate addiction-relevant behaviors in rats and support its further investigation as a treatment for tobacco use disorder.

## Background

Nicotine is the principal addictive component of tobacco [1]. Available pharmacotherapies for the treatment of tobacco use disorder are aimed at modifying the effects of nicotine by either interacting with neuronal nicotinic cholinergic receptors (nicotine replacement therapy, varenicline) or the neurotransmitters mediating nicotine’s effects in the brain (bupropion) [2]. These pharmacotherapies have been helpful for enhancing smoking cessation rates, but most quit attempts still end in failure [3]. New, more effective therapeutic strategies for modifying nicotine’s effects on the brain are therefore of interest. One such approach is the use of nicotine vaccines to bind nicotine in blood and reduce its distribution to brain [4]. This pharmacokinetic strategy showed strong proof-of principle in animals but failed Phase III clinical trials when evaluated by intention-to-treat analysis (all subjects included) [5]. However, enhanced smoking cessation rates were observed in several nicotine vaccine studies in the subset of subjects with the highest antibody concentrations in blood [6, 7]. This finding suggests that a pharmacokinetic approach with sufficient potency could have merit provided that the magnitude of effect on reducing brain nicotine levels is adequate.

An alternative pharmacokinetic strategy being investigated is a nicotine-degrading enzyme that can rapidly reduce nicotine concentrations in blood and nicotine delivery to brain [8, 9]. It has been known for over 60 years that some bacteria living in proximity to tobacco plants can degrade nicotine [10]. The pathways responsible have been identified [11–13] and several of the enzymes involved have been cloned and expressed in purified form [8, 14]. One such enzyme, NicA2 isolated from *P. putida*, can use nicotine as its sole carbon and nitrogen source [12]. It has been proposed [8] that NicA2 degrades nicotine through flavin-dependent catalytic oxidation to methylmyosmine, which is further hydrolyzed to pseudooxynicotine (PON). This pathway is distinct from that of nicotine metabolism in humans, where the conversion of nicotine to cotinine via CYP450 enzymes accounts for 80–90% of endogenous nicotine metabolism. The remainder is metabolized via minor pathways including conversion through 2′-hydroxynicotine to PON [15]. NicA2 mimics this minor pathway. Thus, smokers or users of tobacco products are already chronically exposed to PON and its metabolic intermediates. An initial study of PON safety in rats showed no adverse effects after 5 weeks of administration [8]. Among nicotine’s metabolites in humans only nornicotine is known to share its addictive properties [16, 17]. Degradation of nicotine to PON via NicA2 is therefore an attractive strategy for enhancing nicotine degradation and thereby reducing its effects.

Preliminary studies of NicA2 have characterized the in vitro properties of this enzyme pertinent to its potential therapeutic use [8]. NicA2 is a 52.5 kDa protein which, when expressed in *E. coli*, is complexed with flavin adenine dinucleotide (FAD, a redox co-factor) as indicated by the recently published high-resolution crystal structure [18], and remains catalytically active after isolation without addition of any other components. NicA2 has high catalytic activity with *k*_cat_ of 0.013 s^− 1^, *K*_m_ of 0.092 μM, and *k*_cat_/*K*_m_ = 1.4 × 10^5^ s^− 1^ • M^− 1^ (37 °C), and it rapidly degrades nicotine in vitro at nicotine concentrations representative of serum concentrations in heavy smokers [8].

In a recent report [9], these initial findings have been extended showing that an N-terminal 50-residue truncated form of NicA2 fused to an albumin binding domain (NicA2-J1) demonstrated a prolonged half-life. Pretreatment of rats with this enzyme substantially reduced nicotine distribution to brain. Pretreatment with the enzyme also reduced signs of withdrawal following a 1-week s.c. infusion of nicotine. To further explore the therapeutic potential of enzymatic degradation of nicotine NicA2 was administered to rats to establish its effects on nicotine concentrations in blood and brain over a range of NicA2 doses with both single and repeated doses of nicotine. In addition, we examined its effects on nicotine discrimination and self-administration, models of nicotine addiction widely used to evaluate pharmacotherapies for nicotine or tobacco use disorder.

## Results

### In vitro characterization of NicA2-albumin-binding domain fusion

**Fig. 1:**
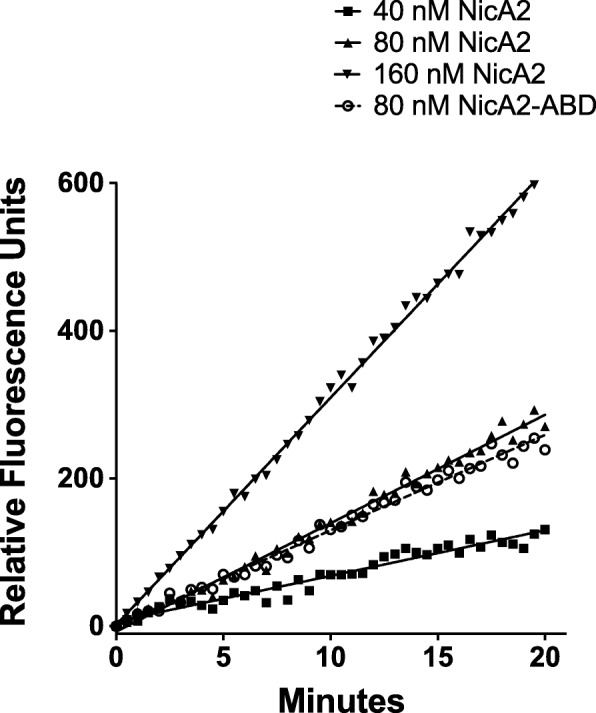
Activity of NicA2 and NicA2-ABD measured by Amplex Red assay

Final purity was > 95% (visual estimate based on SDS-PAGE), with an endotoxin level of < 0.25 EU/mg. The in vitro activity in the Amplex Red assay was indistinguishable between NicA2 and NicA2-ABD (Fig. 1).

### NicA2 selectivity in vitro

NicA2 showed 40–49% activity, compared to nicotine, toward the nicotine metabolite nicotine-1’-N-oxide and the minor tobacco alkaloids nornicotine and anatabine, and no measurable activity toward the remainder of tested compounds (Table 1).

**Table 1:** NicA2 substrate specificity. Activity using the various compounds as substrates measured in an Amplex Red assay. Activities listed in percentages relative to that of nicotine. GABA; γ-amino-N-butyric acid, NAD; β-nicotinamide adenine dinucleotide

Compound	% Activity
Nicotine	100
Anatabine	49
Nicotine-1’-N-oxide	44
Nornicotine	40
Anabasine	< 5
Myosmine	< 5
Norepinephrine	< 5
Dopamine	< 5
Serotonin	< 5
GABA	< 5
Choline	< 5
Acetylcholine	< 5
Nicotinamide	< 5
NAD	< 5
L-Glutamate	< 5
Cytisine	< 5
Mecamylamine	< 5
Varenicline	< 5
Bupropion	< 5

### Completeness of quenching of NicA2 activity by MeOH in vitro

**Fig. 2:**
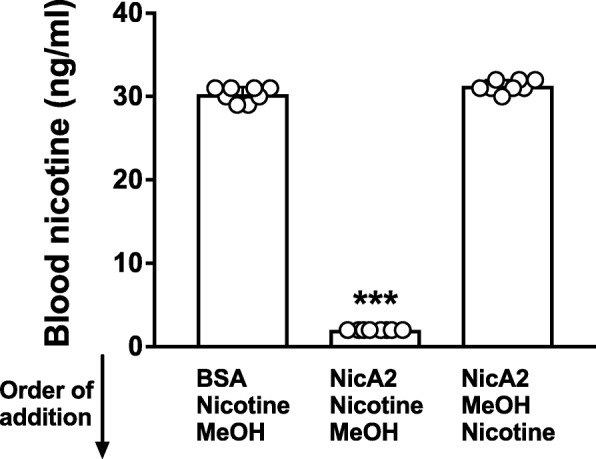
Quenching of NicA2 activity in blood in vitro by MeOH. MeOH was added in vitro to blood containing 40 ng/ml nicotine before or after adding NicA2. Prior addition of MeOH completely quenched NicA2 activity (no difference from BSA control). In contrast, adding MeOH after an approximately 10 s exposure of nicotine to NicA2 in vitro allowed degradation of nicotine to below the limit of assay detection. *** *p* < 0.001 using two-tailed unpaired t tests with Welch’s correction. Mean ± SD, *n* = 8/group

**Fig. 3:**
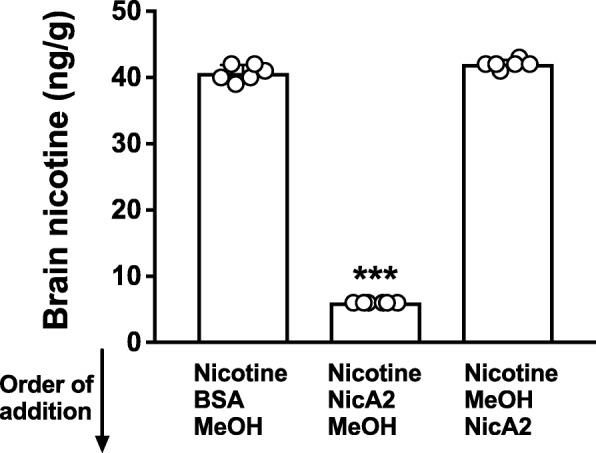
Quenching of NicA2 activity in brain in vitro by MeOH. Addition of MeOH to brain homogenate containing NicA2 prevented the degradation of subsequently added nicotine. However, a 10 s delay in adding MeOH to brain containing nicotine and NicA2 was sufficient to allow substantial degradation of nicotine. *** *p* < 0.001 using two-tailed unpaired t tests with Welch’s correction. Mean ± SD, *n* = 6/group

The ability of MeOH to quench NicA2 activity was tested in vitro by addition of NicA2, nicotine and MeOH in various sequences to blood or homogenized brain. Adding MeOH before mixing NicA2 and nicotine completely quenched NicA2 activity, yielding blood nicotine concentrations that did not differ from those of the BSA control (Fig. 2). In contrast, a delay of approximately 10 s in adding MeOH after mixing NicA2 and nicotine resulted in substantial degradation of nicotine, to below the 2 ng/ml limit of assay detection. Based on these results, in subsequent experiments all blood samples from animals were immediately placed in 4 volumes of methanol and vortexed prior to processing for measurement of nicotine concentrations. Similar results were obtained with brain homogenates and all brain samples in subsequent experiments were homogenized in MeOH (Fig. 3).

### Comparison of NicA2 quenching from in vivo studies via immediate homogenization of brain in methanol v. Flash freezing brain prior to addition of MeOH

There was no difference in measured nicotine concentrations in brain between the group immediately homogenized in MeOH and the group flash frozen and stored prior to addition of methanol (86.2 ± 3.4 ng/g v. 84.6 ± 6.0 ng/g, Mean ± SD, *p* > 0.4).

### Comparison of quenched v. Non-quenched brain from in vivo studies

**Fig. 4:**
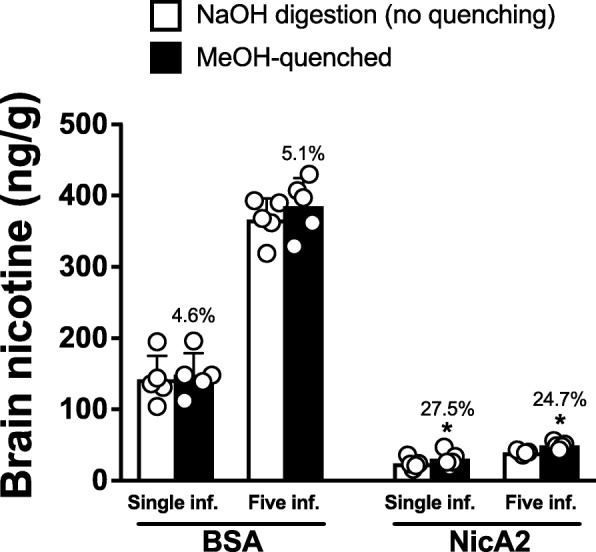
Determining the need to quench NicA2 activity in brain tissue. Brains from the Repeated Nicotine Dose experiment (see Fig. 7 for main result) were split so that one hemisphere was homogenized in MeOH prior to extraction and processing while the other half was not. In animals pretreated with BSA, homogenization in MeOH did not affect the measured nicotine concentrations. In rats pretreated with NicA2, the hemispheres that were not homogenized in MeOH had significantly lower nicotine concentrations, confirming the need to include this step when processing brain tissue. Percentages above bars are the difference between unquenched and quenched groups. * *p* < 0.05, two-tailed paired t tests. Mean ± SD, *n* = 5/group

In control animals receiving BSA prior to nicotine, the measured nicotine concentration did not differ between those processed with or without homogenization in MeOH (Fig. 4). For rats pretreated with NicA2, brain nicotine levels were 27.5% lower (after a single nicotine dose, *p* < 0.05) or 24.7% lower (after multiple nicotine doses, *p* < 0.05) when the MeOH step was omitted. Based on these data all brain tissue in subsequent experiments was rinsed in MeOH and immediately homogenized in 4 volumes of MeOH immediately after removal.

### NicA2 and NicA2-ABD pharmacokinetic parameters

**Fig. 5:**
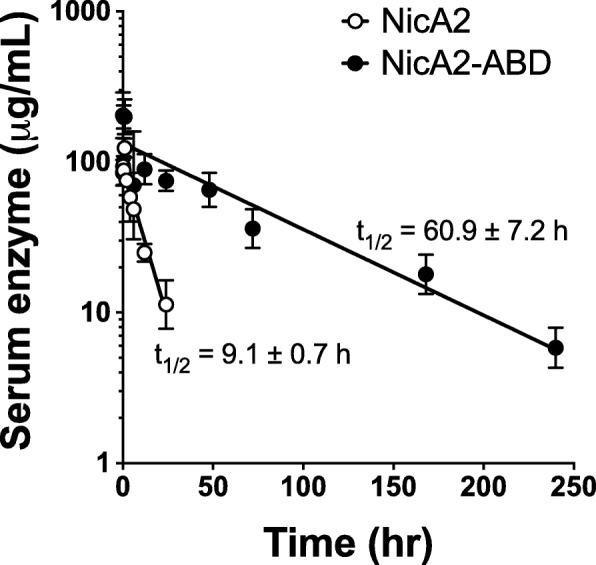
NicA2 and NicA2-ABD pharmacokinetics. Rats (*n* = 3/group) received either NicA2 or NicA2-ABD 5 mg/kg i.v. The terminal half-lives were determined by noncompartmental analysis. Data are the mean ± SD of individual analyses

Enzyme concentration in serum was measured at intervals up to 24 h for NicA2 and 10 days for NicA2-ABD after IV dosing (Fig. 5). Parameters estimated by noncompartmental analysis of NicA2 concentrations include a serum half-life of 9.1 ± 0.7 h, clearance of 0.083 ± 0.015 ml/min/kg, mean residence time of 11.6 ± 1.9 h, and steady state volume of distribution of 0.057 ± 0.005 L/kg. For NicA2-ABD, parameters estimated by noncompartmental analysis include a serum half-life of 60.9 ± 7.2 h, clearance of 0.009 ± 0.002 ml/min/kg, mean residence time of 80.7 ± 6.1 h, and steady state volume of distribution of 0.043 ± 0.006 L/kg. All data are represented as mean ± SD.

### NicA2 effects on blood and brain nicotine levels: Single nicotine dose

**Fig. 6:**
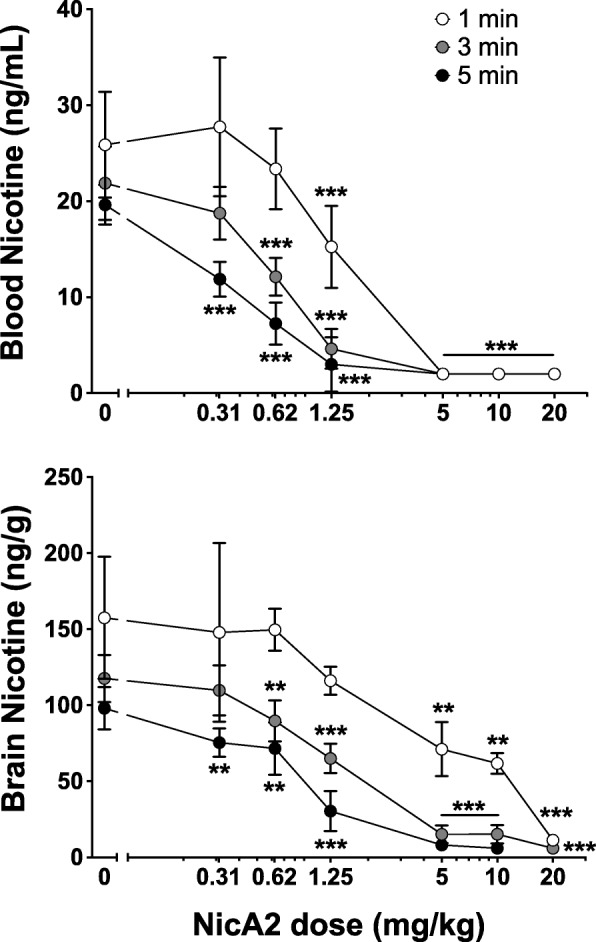
Reduction of blood and brain nicotine concentrations by NicA2. Rats were pretreated with NicA2 i.v. and 5 min later received nicotine 0.03 mg/kg i.v. Groups of rats had nicotine levels measured at 1, 3 or 5 min. Blood (upper panel) and brain (lower panel) nicotine concentrations were reduced by NicA2 in a dose- and time-related manner, with substantial NicA2 effects at doses of ≥5 mg/kg, and with greater reduction of nicotine concentrations at 3 and 5 min than at 1 min. **p < 0.01, ***p < 0.001 compared to BSA using Bonferroni-corrected Welch’s t tests. Mean ± SD, *n* = 8/group

The effects of a range of NicA2 doses on nicotine distribution to blood and brain, over periods of 1, 3 or 5 min, were analyzed (Fig. 6). NicA2 effects were dose and time dependent (*p* < 0.0001 by 2-way ANOVA). NicA2 effects on blood or brain nicotine concentrations were substantial even at 1 min but were greater, particularly in brain, at 5 min.

Blood nicotine levels were significantly lower than in controls at all sampling times in groups receiving NicA2 at doses ≥1.25 mg/kg and were reduced to < 2 ng/ml in all 64 rats receiving NicA2 doses of ≥5 mg/kg. For rats receiving ≥5 mg/kg NicA2 the blood nicotine level was reduced by > 90% at all sampling intervals compared to controls.

NicA2 efficacy in reducing brain nicotine levels was greater at 5 min than at earlier intervals. Brain nicotine levels were significantly lower than controls at 5 min in all groups receiving ≥0.31 mg/kg NicA2, at 3 min in groups receiving ≥0.62 mg/kg NicA2, and at 1 min in rats receiving ≥5 mg/kg NicA2. Although ≥5 mg/kg NicA2 reduced brain nicotine levels by 95% at 3 and 5 min, a higher dose of 20 mg/kg dose was needed to reduce brain nicotine levels to the same extent at 1 min.

### NicA2 effects on blood and brain nicotine levels: Multiple nicotine doses

**Fig. 7:**
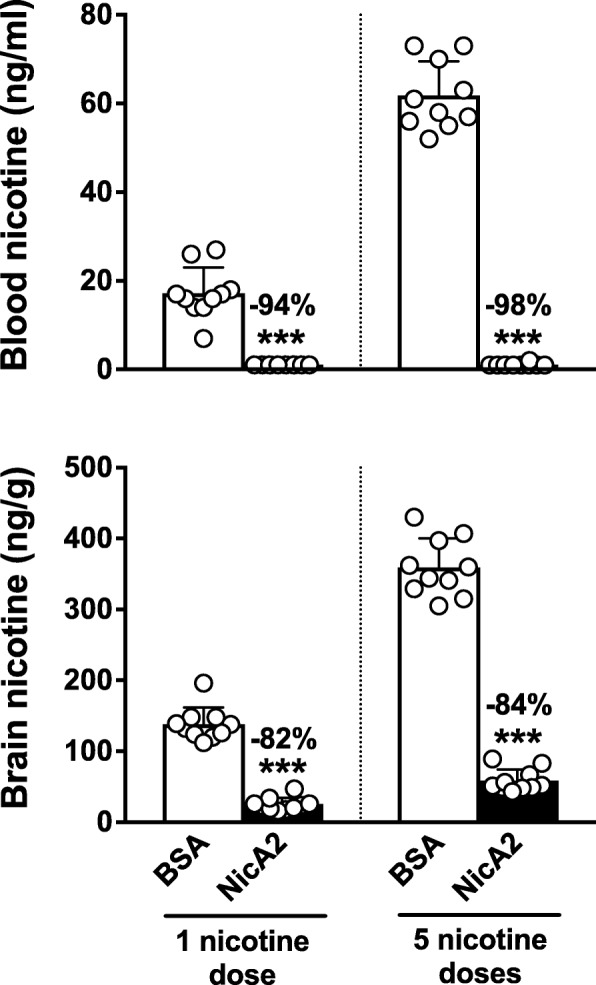
Effects of NicA2 in rats receiving multiple nicotine doses. Rats were pretreated with either 10 mg/kg NicA2 or BSA i.v. Five minutes later two groups received a single nicotine dose of 0.03 mg/kg i.v. and two groups received 5 nicotine doses at 10 min intervals. Blood and brain concentrations were measured 3 min after nicotine dosing. Numbers above bars are the percent reduction of nicotine concentrations compared to BSA control, in blood (upper panel) and brain (lower panel). *** *p* < 0.001, two-tailed unpaired t tests with Welch’s correction. Mean ± SD, *n* = 10/group

Nicotine concentrations in blood and brain were significantly and substantially lower than controls in NicA2-treated rats receiving either a single nicotine dose or a series of 5 nicotine doses (Fig. 7). Blood nicotine concentrations were below the limit of assay detection for most rats receiving NicA2. Brain nicotine concentrations were reduced in rats receiving NicA2 by 82% after the single nicotine dose and by 84% after the series of 5 nicotine doses, compared to their controls.

### Attenuation of nicotine discrimination by NicA2

**Fig. 8:**
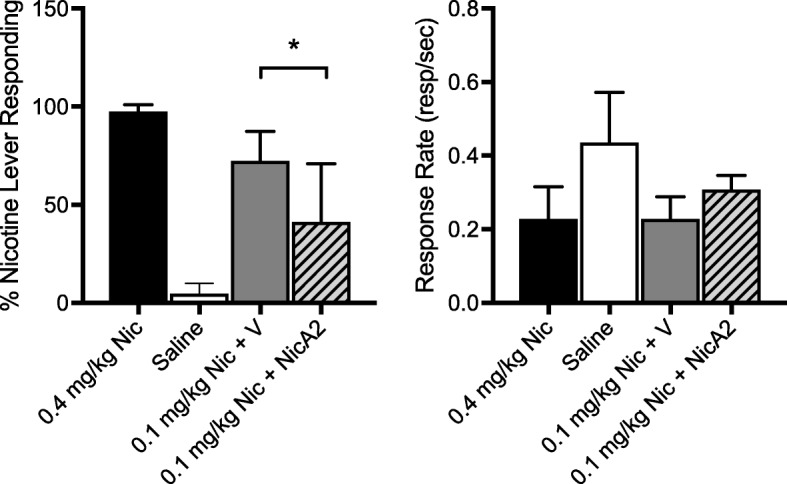
Effect of NicA2 on nicotine discrimination. Each bar represents mean (± SD) percent responding on the nicotine lever (left panel) or overall response rate (right panel) following administration of saline, the training dose (0.4 mg/kg), or the substitution dose (0.1 mg/kg) after PBS vehicle (V) or NicA2 pretreatment. * *p* < 0.05, paired t test (*n* = 4)

Baseline discrimination performance (left panel) and overall response rate (right panel) following the 0.4 mg/kg nicotine training dose and the effects of NicA2 on discrimination during substitution tests with 0.1 mg/kg nicotine are shown in Fig. 8. Substitution of the 0.1 mg/kg nicotine dose following PBS vehicle pretreatment resulted in partial substitution (72.3 ± 15.15 SD % responding on the nicotine-appropriate lever) for the training dose. Following NicA2 pretreatment, percentage of responding on the nicotine-appropriate lever (%NLR) was significantly reduced compared to vehicle (41.1 ± 29.85 SD %NLR, *t* = 3.36, *p* < 0.05). There were no significant effects of NicA2 on response rate, although the higher response rate following saline versus 0.4 mg/kg nicotine and following 0.1 mg/kg nicotine + NicA2 versus 0.1 mg/kg nicotine + vehicle approached significance (*p* = 0.06 and 0.07, respectively). The response rate data indicate that the decrease in %NLR was not simply due to nonspecific suppression of motor activity.

### Attenuation of nicotine self-administration by NicA2

**Fig. 9:**
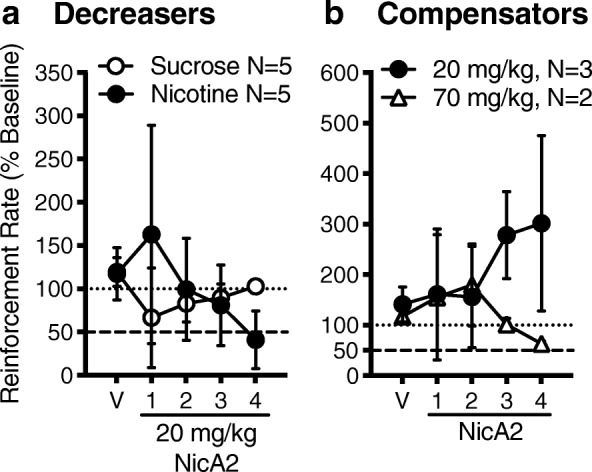
NicA2 effects on nicotine or sucrose self-administration. Mean (± SD) nicotine self-administration (infusion rate) and sucrose-maintained responding (pellet delivery rate) following pretreatment with PBS vehicle (V) and NicA2 over four consecutive test sessions, expressed as a percentage of baseline. A total of 8 rats responding for nicotine were treated with NicA2. Of these, 5 showed a decrease in NSA (Panel **a**; Decreasers) and 3 showed an increase in NSA (Panel **b**; Compensators). Two of these “compensators” were allowed to re-establish baseline NSA and were then treated with 70 mg/kg NicA2 (Panel **b**). Five additional rats responded for sucrose and received NicA2 (Panel **a**). The dotted horizontal line represents baseline. The dashed horizontal line represents the 50% reduction criterion for extinction

The purpose of this experiment was to examine the ability of NicA2 to block the reinforcing effects of nicotine in a self-administration model. Figure 9 shows nicotine self-administration (infusions earned) and sucrose-maintained responding (response rate) following vehicle pretreatment and NicA2 pretreatment over four consecutive test sessions. Pretreatment with PBS vehicle did not alter responding in either the nicotine or sucrose group (Panel A and B). Although there was no statistically significant effect of NicA2 when data from all eight rats were analyzed together, there was a clear dichotomy in the pattern of NSA between rats. Responding for nicotine decreased over the four NicA2 treatment sessions in five of eight rats (Panel A; Decreasers). In these five rats NSA decreased by 65% by day four compared to vehicle. Four of these rats showed a brief increase in NSA on the first day of NicA2, similar to the extinction burst that occurs in some rats when saline is substituted for nicotine [19]. In contrast to the 5 Decreasers, 3 of the eight NSA rats exhibited a compensatory increase in NSA with the 20 mg/kg NicA2 dose (Panel B; Compensators). However, increasing the NicA2 dose to 70 mg/kg in two of these animals decreased NSA by 37% by day four (Panel B). The third rat’s catheter failed before the higher NicA2 dose could be tested. Pretreatment with NicA2 did not affect responding for sucrose compared to vehicle (Panel A). Two rats showed a significant decrease on day 1 of NicA2 treatment but returned to baseline across days 2 to 4.

**Fig. 10:**
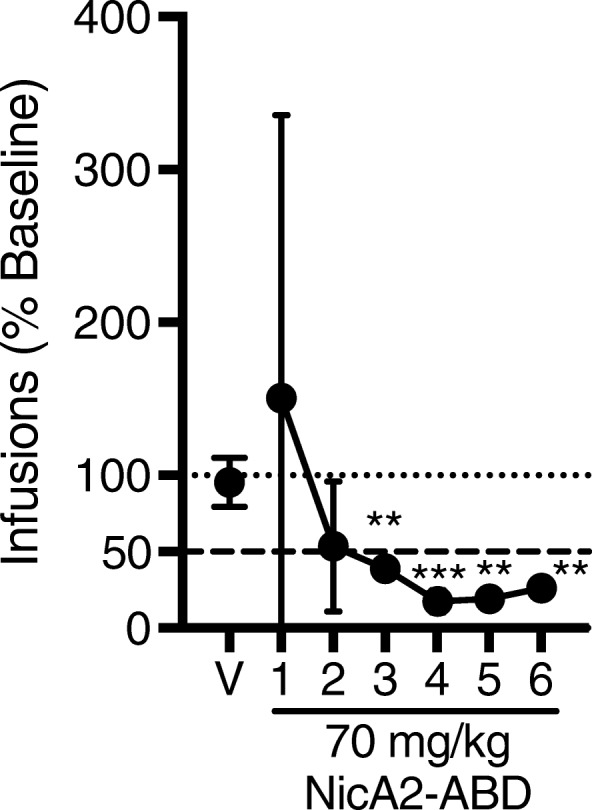
Effects of NicA2-ABD on NSA during unlimited access to nicotine. Mean (± SD) number of infusions during 23-h access following pretreatment with PBS vehicle (V) and NicA2-ABD over six consecutive test sessions, expressed as a percentage of baseline. Each point represents the mean of four rats. The dotted horizontal line represents baseline. The dashed horizontal line represents the 50% reduction criterion for extinction. Different from V, **p < 0.01, ***p < 0.001

Figure 10 shows nicotine self-administration (infusions earned) following vehicle pretreatment and NicA2-ABD pretreatment (70 mg/kg i.v.) over six consecutive test sessions for four rats. PBS vehicle did not alter NSA, whereas NSA decreased over the six daily NicA2-ABD treatment sessions in all rats, with NSA significantly reduced by 74% by day six compared to vehicle (*t* = 6.99, *p* < 0.01). One rat exhibited an extinction-like increase in NSA only on the first day of NicA2-ABD.

## Discussion

The main findings from the present assessment of NicA2 activity in vivo are that pretreatment of rats with NicA2: 1) markedly reduced the early distribution of nicotine to brain when nicotine was administered as a single rapid i.v. bolus dose, 2) reduced nicotine distribution to brain when nicotine was administered as repeated i.v. doses at a mg/kg rate comparable to heavy cigarette smoking, and 3) attenuated nicotine discrimination and nicotine reinforcement, effects that are predictive of the efficacy of smoking cessation medications. In addition, NicA2′s elimination half-life of 9.1 h in rats was increased to 60.9 h by fusion with an albumin binding domain without impairing its catalytic activity. These data suggest that the enzymatic degradation of nicotine via NicA2, with further optimization of its catalytic activity and attenuation of immunogenicity, is of interest as a potential smoking cessation medication.

An analogous approach has been explored with the use of cocaine-degrading enzymes for the treatment of cocaine use disorder or overdose. Experience with these enzymes is limited but helpful in assessing the therapeutic potential of NicA2. Two enzymes have shown considerable activity in clinical laboratory studies. A mutated bacterial enzyme RPB-8000, being developed as a treatment for cocaine overdose, was administered to human subjects at a dose of 200 mg/kg 1 min after an i.v. cocaine dose of 50 mg/kg. This enzyme reduced the serum cocaine concentration by 90% within 2 min and reduced total exposure to cocaine (area under the time-concentration curve) by 95% [20]. An engineered variant of human butyryl cholinesterase (Alb-BChE; TV-1380), with increased catalytic activity and a longer serum elimination half-life than the wild-type, is also being developed as a potential therapeutic agent for cocaine use disorder. Pretreatment of human subjects with 100–300 mg/kg Alb-BChE reduced the peak plasma cocaine concentration and elimination half-life following an i.v. cocaine dose of 40 mg/kg by > 80%, as well as its subjective effects [21]. These studies are consistent with preclinical data showing that a drug-degrading enzyme strategy can be effective in rapidly and substantially reducing cocaine plasma levels and attenuating its effects [22]. The high doses of these enzymes needed, however, have limited their clinical development.

The cocaine-degrading enzyme data are of particular interest because the single and daily doses of cocaine that are typically abused are more than an order of magnitude higher than those of nicotine delivered to cigarette smokers. Single cocaine doses of up to 40 mg (approximately 0.6 mg/kg) have been administered i.v. in clinical laboratory studies, with delivery of up to 7 doses of this size, or about 4 mg/kg over a 2.5 h session [23]. Self-reported doses of cocaine abused outside of a controlled setting may be considerably higher [24]. In contrast, one cigarette typically delivers about 1 mg (0.014 mg/kg) of nicotine over 5–10 min as 5–10 divided doses (puffs), about 3% the size of a typical laboratory cocaine dose [25]. A one pack per day smoker will receive about 20 mg of nicotine over 12–16 h, or 0.29 mg/kg. This is about 7% of a potential cocaine laboratory session total dose. All else being equal, an enzyme-based drug lowering strategy should be more feasible for nicotine, since the amount of drug to be metabolized is considerably smaller and its rate of delivery considerably slower.

While the catalytic efficiency of NicA2 (*k*_cat_/*K*_m_ of 1.4 × 10^5^ s^− 1^ M^− 1^) [8] is lower than that of Alb-BChE (*k*_cat_/*K*_m_ = 2.3 × 10^7^ s^− 1^ M^− 1^) [26] the NicA2 enzyme, unlike Alb-BChE has not yet undergone optimization to enhance its activity. More importantly, even at its current level of catalytic activity, NicA2 is highly effective in preventing nicotine from reaching brain in rats when nicotine is administered at single or multiple doses exceeding those received by heavy smokers. The effects of NicA2 were somewhat greater at 5 min after a nicotine dose than at 1 min, but were nevertheless substantial at 1 min. It is possible that the very rapid i.v. bolus delivery of nicotine in the current study, at doses higher than are delivered by a single puff of a cigarette, overwhelmed the catalytic capacity of the enzyme at 1 min, and that NicA2 would prove more effective if nicotine were delivered to rats in smaller incremental doses, as in smokers. In support of this possibility, NicA2 blocked nicotine discrimination when the nicotine dose of 0.1 mg/kg was even larger than in the pharmacokinetic studies but was administered s.c. rather than i.v. and therefore absorbed more slowly.

Consistent with the effects of NicA2 on nicotine distribution to brain, NicA2 attenuated nicotine discrimination and decreased nicotine reinforcement in a nicotine self-administration model while it had no effect on responding for sucrose, i.e. NicA2’s effects were specific for nicotine. Although the reinforcing effect of nicotine was attenuated in all rats, it was manifested in two distinct patterns. At the 20 mg/kg NicA2 dose most rats showed a moderate increase (extinction burst) in NSA, followed by a decrease to extinction-like levels by day 4, while other rats showed only a compensatory increase in NSA, presumably to surmount decreased brain nicotine levels. However, this compensatory response was avoided by increasing the NicA2 dose. These data suggest that 20 mg/kg is a near-threshold effective dose. The longer half-life of the NicA2-ABD fusion construct allowed it to be evaluated in a 23 h/day access NSA model, in which nicotine dosing more closely resembles the nicotine exposure of a smoker, and attenuation of nicotine reinforcement was confirmed.

Recently reported studies of a similar NicA2 albumin-binding domain enzyme fusion (NicA2-J1) [9] are consistent with and complementary to the current findings. When this enzyme was administered to rats during a 7-day nicotine infusion, it reduced signs of withdrawal following termination of the nicotine infusion compared to controls. Brain nicotine levels were below the limit of detection in the rats treated with NicA2-J1. This study supports the feasibility of extending NicA2’s half-life to prolong its duration of effect.

The current study extends these findings by providing a) enzyme dose-response data over a range of time-frames, b) showing substantial enzyme effects on nicotine distribution to brain when nicotine is administered repeatedly, as i.v. boluses at doses comparable to heavy smoking c) showing effects of NicA2 on the key behavioral measures of nicotine discrimination and reinforcement, and d) confirming that extending NicA2’s half-life via an albumin binding domain fusion does not affect its catalytic activity. It is noteworthy that NicA2 substantially blocked nicotine distribution to brain or its behavioral effects when nicotine was administered as large i.v. bolus doses. This method of nicotine delivery mimics the rapid uptake kinetics of nicotine from smoking [27].

Further support that a pharmacokinetic intervention could have therapeutic potential for smoking cessation comes from studies of nicotine vaccines and nicotine-specific monoclonal antibodies, both of which can reduce nicotine distribution to brain in animals and block addiction-relevant behaviors [4]. NicA2 is likely to be more potent than a nicotine vaccine or monoclonal antibody. In the current study, pretreatment of rats with NicA2 10 mg/kg resulted in an 82% reduction in the distribution of a single nicotine dose to brain. In a previously reported study of the high affinity nicotine-specific monoclonal antibody Nic311 which used a similar protocol (nicotine 0.03 mg/kg i.v. and measurement of brain nicotine level 3 min after the dose), Nic311 80–160 mg/kg was required to produce a comparable reduction in nicotine distribution to brain [28]. In addition, NicA2 remained highly effective in reducing brain nicotine levels after 5 nicotine doses delivered over 40 min (> 80% reduction in brain nicotine level compared to controls), while previous studies of a nicotine vaccine showed that its effects were considerably smaller after the same cumulative nicotine dose (< 30% reduction) [29]. Nicotine vaccines have shown signals of efficacy in clinical trials of smoking cessation with higher smoking cessation rates, albeit only in the subset of subjects that achieved the highest serum antibody concentrations [6, 7]. If NicA2 proves more potent than nicotine-specific antibodies or vaccines in humans for reducing nicotine distribution to brain, as it appears to be in rats, it could also be more effective for enhancing smoking cessation rates.

A methodologic finding from this study is the importance of rapidly quenching blood or brain with MeOH to terminate NicA2 activity ex vivo. For blood, even a 10 s delay in doing so markedly reduced measured nicotine concentrations. Effects of quenching on brain nicotine levels were more limited, although still significant, presumably because little NicA2 enters brain tissue and only enzyme remaining in brain blood vessels needs to be quenched.

Several important hurdles must be addressed for NicA2 to be a useful therapeutic agent. Its serum elimination half-life needs to be lengthened to allow a suitable dosing frequency. The strategy of prolonging the enzyme’s duration of action through fusion with an albumin-binding domain may be sufficient, as the serum half-life of albumin in humans is 3 weeks [30] but further study is needed to explore this option. A bacterial protein may be immunogenic and this potential must be minimized. Fortunately, the expected duration of treatment with enzyme needed for smoking cessation is relatively brief, typically 12 weeks based on experience with nicotine replacement therapy, bupropion and varenicline [2]. This relatively short expected duration of treatment should reduce the opportunity for development of anti-NicA2 antibodies. In addition, it would be desirable to further increase the catalytic activity of NicA2 both to enhance its effectiveness and to lower the required dose.

Components of tobacco or tobacco smoke, other than nicotine, are behaviorally active in various animal models [17, 31, 32]. Tobacco alkaloids including anabasine, anatabine and myosmine are present at much lower concentrations than nicotine and are also far less potent as reinforcers. Their contributions to tobacco use disorder or addiction, if any, are likely minimal [32, 33]. Acetaldehyde is reinforcing in rodents [17] but at doses considerably higher than those delivered by smoking [34]. Tobacco pH may influence smoking behavior but does so by modifying nicotine absorption [1]. Monoamine inhibitors present in tobacco can modify nicotine’s effects and enhance nicotine self-administration in rats but are not by themselves reinforcing [35]. In contrast, the evidence that the nicotine content of cigarettes drives tobacco use is abundant [1, 36]. Reducing a smoker’s exposure to nicotine using an enzyme such as NicA2, in order to promote smoking cessation, has a strong rationale.

Limitations of this study include the use of only one nicotine dose size in the pharmacokinetic experiments, and relatively small groups sizes in the behavioral studies. NicA2 had no obvious adverse effects in this study, aside from the decrease in sucrose-maintained responding in two rats on the first day of treatment but examining enzyme safety per se was not a specific goal. NicA2 showed high catalytic activity toward nicotine and, to a lesser extent, the nicotine metabolite nicotine-N-oxide and the minor tobacco alkaloids nornicotine and anatabine, but did not metabolize any of the endogenous ligands or other compounds examined. A previous study showed no adverse effects of 5 weeks of dosing with pseudooxynicotine, the primary metabolite of nicotine through the action of NicA2. This is not surprising since pseudooxynicotine is a normally present minor metabolite of nicotine in humans [15]. Further studies of the safety of optimized versions of NicA2 will of course be needed.

## Conclusions

NicA2 rapidly reduced blood and brain nicotine concentrations when administered to rats at single or multiple nicotine doses relevant to the nicotine intake of cigarette smokers. NicA2 also reduced the reinforcing potency of nicotine in a rat model of nicotine self-administration. These data establish NicA2 as a promising starting point for further optimization and development as a therapeutic agent and a novel treatment strategy for tobacco use disorder.

## Methods

### NicA2 preparation and in vitro characterization

NicA2 for in vitro studies was generated as described in [8]. For in vivo experiments, a similar expression construct was generated by cloning a synthetic gene encoding the same wildtype NicA2 amino acid sequence (GenBank: AEJ14620.1) [14] with an added C-terminal His_6_-tag (optimized for *E. coli* expression; GeneArt/Invitrogen) into pET22b(+), and was transformed into the *E. coli* expression strain BL21(DE3) (Agilent).

Purification of enzyme for in vivo testing added steps for endotoxin removal including 0.1% of the non-ionic surfactant octylphenol ethoxylate (Triton X-114; Sigma-Aldrich, St. Louis, MO) in the wash buffer during cobalt immobilized metal affinity chromatography purification (using Talon resin; Clontech), followed by tangential flow filtration buffer exchange and an additional polishing step in the form of anion exchange chromatography using a Q Sepharose FF column (GE Life Sciences). Fractions containing NicA2 (by SDS-PAGE/Coomassie stain) were pooled, dialyzed into PBS pH 7.4 and concentrated. Concentration was determined by UV absorbance at 280 nm using the theoretically determined extinction coefficient A_280_ at 1 g/L = 1.313 [37]. Endotoxin levels were determined using an Endosafe® PTS™ instrument (Charles River). Final purity was > 95% (visual estimate based on SDS-PAGE), with an endotoxin level of 0.12 EU/mg.

Activity of purified protein was measured in vitro using the Amplex Red assay kit (Thermo). Based on NicA2’s proposed mechanism, oxidation of nicotine results in the generation of H_2_O_2_ which is coupled to the conversion of the colorless Amplex Red reagent into its red-fluorescent product, resorufin by horseradish peroxidase [38]. Assays were performed as per the manufacturers protocol, including S-(−)-nicotine (Sigma) at a final assay concentration of 10 μM in 96-well black half-area flat bottom plates (Corning). Fluorescence was detected in a SpectraMax M2 plate reader using excitation at 555 nm, detection at 590 nm, and employing a “Plate Blank” well to subtract the value derived from the no enzyme control for each point in the SoftMax® Pro data evaluation software package (Molecular Devices). Activities were expressed as the relative slopes of increase in fluorescence as a function of time. Development of fluorescence was dependent on the presence of nicotine, and the rate of fluorescence-development was proportional to the concentration of NicA2 in the range used.

### Substrate specificity of NicA2

Substrate specificity of NicA2 was analyzed by the Amplex Red assay described above, using 10 μM of test compound and 160 nM NicA2 enzyme. Compounds tested were: (2’S)-nicotine-1’-N-oxide (Toronto Research Chemicals; TRC), (±)-nornicotine, nicotinamide, β-nicotinamide adenine dinucleotide (NAD), acetylcholine, choline, (−)-cotinine, varenicline, bupropion, (−)-cytisine, mecamylamine (TRC), dopamine, serotonin, (±)-norepinephrine, L-glutamate, γ-amino-N-butyric acid (GABA), (R,S)-anatabine (TRC), (R,S)-anabasine (TRC), and myosmine (compounds obtained from Sigma-Aldrich unless otherwise stated). Activities were expressed relative to the activity found for S-(−)-nicotine run in parallel.

### Preparation of NicA2-albumin-binding domain fusion and in vitro characterization

A gene fusion was prepared consisting of the NicA2 amino acid sequence mentioned above fused at its C-terminus to a 5 kDa albumin binding domain (ABD035, which binds albumin with high affinity across rodents, non-human primates and humans [39]) via a flexible Gly_4_Ser linker followed by a C-terminal His_6_-tag (gene optimized for *E. coli* expression; GeneArt/Invitrogen). This construct was cloned into pET22b(+) and transformed into the *E. coli* expression strain BL21(DE3) (Agilent). Expression and purification was carried out as described above.

### Nicotine assay and quenching of NicA2 activity

Nicotine concentrations in blood or brain were measured using gas chromatography with nitrogen phosphorus detection [40, 41]. Concentrations that were below the limit of quantitation for the assay were considered to be at the limit of 2 ng/ml for purposes of analysis. For this assay blood undergoes solvent extraction, while brain is first digested in NaOH before extraction. Because residual NicA2 in samples could continue to degrade nicotine ex vivo, blood samples were quenched immediately upon collection by drawing blood into a tube and transferring 0.5 ml into 4 volumes of methanol and immediately vortexing.

Completeness of quenching of NicA2 activity in blood by MeOH was assessed in vitro by comparing samples prepared by adding the following to 0.5 ml blood, in this order: **a)** BSA 100 μg/ml **→** nicotine 40 ng/ml **→** 4 volumes of methanol; **b)** NicA2 100 μg/ml (the approximate blood concentration of NicA2 following a 10 mg/kg i.v. dose) **→** nicotine 40 ng/ml **→** 4 volumes of methanol; **c)** NicA2 100 μg/ml **→** 4 volumes of methanol **→** nicotine 40 ng/ml. Tubes were vortexed for 5 s at each step. Samples were stored at − 20 °C until assayed for nicotine levels. A similar protocol was performed using brain homogenate containing 40 ng/ml nicotine in place of blood, to determine completeness of quenching of brain samples by MeOH in vitro. Nicotine concentrations were compared using two-tailed unpaired t tests with Welch’s correction and adjusted for multiple comparisons (α = 0.025).

For in vivo studies blood was obtained either through an indwelling venous catheter or as trunk blood after decapitation, and NicA2 activity was quenched as above. Brain was rapidly removed after decapitation, rinsed in methanol, and immediately homogenized with 4 volumes of methanol. At this point brain was stored at − 20 °C with no loss of nicotine concentration. Alternatively, to facilitate storage and shipping of samples, brain could be rinsed, flash frozen in liquid nitrogen and stored at − 80 °C until assayed. When ready for assay, the frozen sample was placed in 4 volumes of methanol and processed as above. The adequacy of flash-freezing brain prior to addition of methanol was evaluated by pretreating 5 rats with 1.25 mg/kg NicA2 and administering nicotine 0.03 mg/kg 5 min later (equivalent to two cigarettes in a human). Brains were collected 3 min after nicotine administration. Brains were rinsed in methanol and one hemisphere of each brain was immediately homogenized in 4 volumes of methanol while the other half was flash frozen and stored at − 80 °C until assayed. Groups were compared with a two-tailed paired t test.

Because it was expected that very little NicA2 would cross the blood-brain barrier owing to its molecular weight of 52.5 kDa, it was initially unclear whether brain, after rinsing in methanol, required further quenching by methanol. This question was addressed by dividing samples obtained as part of the repeated nicotine dose pharmacokinetic experiment described below. In this experiment brain was obtained from rats that had been pretreated with NicA2 and then received either 1 or 5 doses of nicotine. Brains were first rinsed in MeOH and then split so that one hemisphere was processed only by rinsing the whole brain in methanol and the other hemisphere was processed by immediately placing it in 4 volumes of methanol and homogenizing. Groups were compared using two-tailed paired t tests.

### Estimation of NicA2 and NicA2-ABD pharmacokinetic parameters

Female Sprague Dawley rats weighing 225–250 g were obtained with a jugular venous catheter in place (Charles River). The choice of male or female rats in this and subsequent experiments was depending upon their availability and desired weight range at the time of each experiment. An additional goal was to test efficacy of NicA2 in both male and female rats. Three rats received 5 mg/kg His-tagged NicA2 via the tail vein. Blood (0.2 ml) was collected into serum separator tubes via the jugular catheter at pre-dose and over a 5 min-24 h period for NicA2 or 5 min-10 days for NicA2-ABD, and serum was isolated and stored at − 20 °C until analysis. Assay of NicA2 or NicA2-ABD concentrations in serum samples took advantage of the C-terminal His-tag. MaxiSorp ELISA plates (Nunc) were coated overnight with anti-His tag antibody (R&D Systems). Plates were blocked with 1% non-fat dry milk (NFDM) in PBS for approximately 1 h. Dilutions of NicA2 or NicA2-ABD standards (for the latter a pre-incubation step in rat serum was conducted so the standard curve would accurately represent the NicA2-ABD:albumin complex detection in the actual samples) and serum samples in 1% NFDM in PBS + 0.1% Tween-20 were added to the plates and incubated for 2 h at room temperature. After washing away unbound substances (all wash steps performed in PBS + 0.1% Tween-20), rabbit anti-NicA2 polyclonal primary detection antibody (custom reagent generated by Noble Life Sciences) was added to the wells for a 1 h incubation. A wash step was followed by addition of horseradish peroxidase-conjugated goat anti-rabbit IgG (Fc) (KPL International). Plates were washed, and the remaining binding complex was detected with TMB substrate (3,3′,5,5′-tetramethylbenzidine; KPL International). Once stopped with acid, plates were read on a spectrophotometer at 450 nm and data analyzed in SoftMax® Pro, version 5.4 (Molecular Devices). Estimates of pharmacokinetic parameters (volume of distribution, clearance, terminal half-life) were obtained from serum concentrations using noncompartmental methods [42].

### NicA2 effects on blood and brain nicotine levels: Single nicotine doses

Female Sprague Dawley rats weighing 225–250 g were purchased with jugular venous catheters in-place (Charles River Labs). Fifteen groups of 8 rats were pretreated with NicA2 through the catheter with 3 groups at each of the following NicA2 doses: 0.3125, 0.625, 1.25, 5.0, 10.0 mg/kg. Three control groups of 8 rats each received bovine serum albumin 4 mg/kg rather than NicA2. Five min later each group received 0.03 mg/kg nicotine i.v. Six groups of rats (one group at each NicA2 dose and one control group) were then sacrificed at 1, 3 or 5 min following the nicotine dose. Blood and brain samples were obtained by decapitation and quenched with methanol as described above. Two additional groups were pretreated with NicA2 20 mg/kg and studied as above but blood and brain nicotine levels measured only at 1 and 3 min based on pilot data showing blood and brain levels were undetectable at 5 min. Blood or brain nicotine concentrations were compared by Bonferroni-corrected Welch’s t-tests to accommodate heterogeneity of variance between doses or time points. Each time point or dose was considered a separate family of comparisons, such that the significance level was set at *p* = 0.0083 for comparing each NicA2 dose to BSA for each time point. Serum nicotine levels were not normally distributed in two groups and brain nicotine levels were non-normal in one group. This was due to the presence of one outlier in each group, confirmed by Iterative Grubb’s analysis (*p* < 0.01). These outliers were removed for the statistical analyses but were included in the figures.

### NicA2 effects on blood and brain nicotine levels: Multiple nicotine doses

Male Holtzman Sprague Dawley rats weighing 340–470 g were anesthetized with 0.1 mg/kg fentanyl and 0.05 mg/kg dexmedetomidine i.m. and 100 mg/kg propofol i.p. and a jugular venous catheter was placed. Two groups of 10 rats received NicA2 10 mg/kg i.v. via the jugular catheter and two groups received 10 mg/kg BSA as controls. Five min later one NicA2 and one BSA group received nicotine 0.03 mg/kg i.v. These groups were sacrificed 3 min after the nicotine dose, and blood and brain obtained by decapitation. The two remaining groups received nicotine 0.03 mg/kg i.v. every 10 min × 5 and were sacrificed 3 min after the fifth nicotine dose. Blood and brain nicotine concentrations were compared between NicA2 and BSA groups using two-tailed unpaired t tests with Welch’s correction for unequal variances.

### Effect of NicA2 on nicotine discrimination

Procedures were similar to those previously used in our laboratory [43, 44]. Four male Sprague Dawley rats weighing 550–600 g had been trained to discriminate nicotine alone (0.4 mg/kg s.c. from saline using a 2-lever discrimination procedure). Lever pressing was reinforced under a terminal variable-interval 15 s schedule using 45 mg food pellets. Discrimination was assessed twice weekly (Tues and Fri) during 2 min extinction test sessions. Discrimination was considered stable when a) > 80% responding occurred on the injection-appropriate lever during two consecutive saline and nicotine test sessions, b) > 90% injection-appropriate responding occurred on six consecutive training sessions, and c) response rates (total responses/session) were stable (no trend across these four test sessions and six training sessions). When performance was stable, rats were habituated to being placed in restraint tubes for tail vein injection of NicA2 by injecting saline via tail vein 10 min prior to two or more test sessions until restraint had no effect on discrimination performance. At this point, the effect of 10 mg/kg NicA2 i.v. on the ability of 0.1 mg/kg nicotine s.c. to substitute for the 0.4 mg/kg training dose was determined. During these test sessions, PBS or NicA2 was administered i.v. 10 min prior to 0.1 mg/kg nicotine s.c.. The 0.1 mg/kg nicotine s.c. dose was used because it produces serum nicotine concentrations similar to those produced by the 0.03 mg/kg i.v. dose in the pharmacokinetic studies. This dose normally produces partial substitution for a 0.4 mg/kg training dose, i.e. about 50–60% nicotine lever responding [43, 44]. The percentage of responding on the nicotine-appropriate lever (%NLR) and overall response rate (responses/second) during the 2-min extinction test sessions served as the primary dependent measures. These measures were compared between vehicle and NicA2 administration prior to the 0.1 mg/kg nicotine substitution test sessions via paired t-test.

### Effect of NicA2 on nicotine or food self-administration

Procedures were similar to those previously used in our laboratory [32]. A total of 8 male Holtzman Sprague Dawley rats weighing 310–460 g were used. Four were experimentally naïve. One was previously trained to self-administer a unit nicotine dose of 0.06 mg/kg. Three had failed to self-administer anabasine in an unrelated pilot study. Naïve rats were implanted with jugular cannulas 1 week after arrival. One week later the rats were placed in operant conditioning chambers and allowed to acquire nicotine self-administration (NSA) using 2 h sessions at a 0.03 mg/kg unit nicotine dose and gradually escalating the fixed-ratio (FR) schedule to FR 3 over several weeks. Non-naïve rats were similarly trained. On average, the rats self-administered 0.51 mg/kg/day (16.9 ± 4.1 SD infusions) of nicotine over the 2 h sessions. Rats were considered to have acquired NSA when they earned at least 8 infusions per session and responding on the active lever compared to the inactive was > 2:1. After at least 1 week at FR 3, if NSA was stable (< 15% variation and no trend), rats received an i.v. infusion of PBS vehicle 10 min before one session (Mon), followed by i.v. infusion of 20 mg/kg NicA2 10 min before each of four consecutive sessions (Tues-Fri). Rats were then allowed to reacquire NSA until stable. To confirm that these rats were sensitive to changes in nicotine exposure per se, NicA2 effects were compared to extinction of NSA by substituting saline for nicotine for 4 consecutive sessions (Tues-Fri). The order of the extinction and NicA2 treatment phases was counterbalanced across subjects. Another group of 5 rats was trained to respond for sucrose pellets to examine whether NicA2 effects were selective for NSA or produced nonspecific side effects. These rats were trained under the same FR 3 schedule as the NSA group. Sessions ended after 2 h or when 50 pellets were earned. After responding was stable (no trend in response rate over 5 consecutive sessions), rats received an i.v. infusion of PBS vehicle 10 min before one session (Monday), followed by i.v. infusion of 20 mg/kg NicA2 10 min before each of the following four consecutive sessions (Tues-Fri). In two rats, a higher NicA2 dose (70 mg/kg) was then tested 6–12 weeks later in the same manner after recovery of baseline NSA performance and allowing elimination of NicA2. Reinforcement rate (reinforcers/min) was calculated for NSA (infusions delivered) and sucrose self-administration (pellets delivered). This measure for vehicle and NicA2 test sessions was transformed to a percentage of baseline (mean of the week before NicA2 testing), which served as the primary dependent measure. Paired t-tests with a Bonferroni correction for multiple comparisons were used to compare this measure during each NicA2 session to vehicle (*p* < 0.012 for four comparisons) for NSA or sucrose self-administration separately. Effects of the higher NicA2 dose were considered significant if the number of infusions during NicA2 treatment was below the range of infusions during baseline.

A separate group of four male Holtzman Sprague Dawley rats weighing 325–360 g was used in a pilot NSA study to test the longer-acting variant of the enzyme (NicA2-ABD) in rats with 23 h/day access to nicotine. The longer-acting enzyme allowed use of the 23 h nicotine access model which more closely resembles human nicotine exposure than does a 2 h session. Rats were trained to self-administer a unit nicotine dose of 0.03 mg/kg using the same procedures described above, except that sessions were 23 h in duration. Cage maintenance was done during the 1 h between sessions. After NSA was stable (same criteria), saline was substituted for nicotine to extinguish NSA. When the number of infusions decreased by at least 50% and there was no trend across three consecutive session, rats were allowed to reacquire stable NSA. Rats were then given a PBS vehicle infusion 10 min before one session, followed by infusion of 70 mg/kg NicA2-ABD 10 min prior to each of six consecutive sessions (the average number of session required for saline extinction). This dose of NicA2-ABD was used because of the variability in effect observed for the 20 mg/kg NicA2 dose during 2 h sessions and the higher nicotine intake occurring in the 23 h sessions (1 mg/kg/day, 33.3 ± 10.5 SD infusions v. 0.51 mg/kg/day for the 2 h sessions). Data were analyzed in the same way as the 2 h data (*p* < 0.0083 for six comparisons).

## Abbreviations

ABD: Albumin binding domain
BChE: Butyryl cholinesterase
BSA: Bovine serum albumin
FAD: Flavin adenine dinucleotide
FR: Fixed-ratio
GABA: γ-amino-N-butyric acid
NAD: Nicotinamide adenine dinucleotide
NFDRM: Non-fat dry milk
NLR: Nicotine-appropriate lever
NSA: Nicotine self-administration
PON: Pseudooxynicotine
